# Dataset of student level prediction in UAE

**DOI:** 10.1016/j.dib.2021.106908

**Published:** 2021-02-24

**Authors:** Shatha Ghareeb, Abir Hussain, Wasiq Khan, Dhiya Al-Jumeily, Thar Baker, Rawaa Al-Jumeily

**Affiliations:** a Liverpool John Moores University, United Kingdom; b University of Sharjah, United Arab Emirates

**Keywords:** Student grade prediction, Student tracking, Education, Levelling, Artificial intelligence, School curriculum

## Abstract

A primary dataset is presented comprising student grading records and educational diversity information. The dataset is collected from two international schools, a British curriculum, and an American Curriculum schools based in Abu Dhabi, United Arab Emirates. Following the ethical approval from Liverpool John Moores University (19/CMS/001), the data is collected through gatekeepers. A permission letter was granted from the Ministry of Education and Knowledge in Abu Dhabi, UAE to provide access to the schools. The dataset is anonymised by eliminating sensitive and identifiable students’ information and prepared to be used for pattern analysis and prediction of student grading based on diverse educational backgrounds that might be useful for automated student levelling, i.e., at which level the student needs to be entered when moved from a different school with different international curriculum.

## Specifications Table

**Table:** 

Subject	Student level prediction
Specific subject area	Student levelling, school's recommendation in multicultural environments, diversity in education, grade prediction
Type of data	Table (.xlsx)
How data were acquired	The data was collected from a nominated school gatekeeper in order to control what information was given.
Data format	Raw data (.xlsx)
Parameters for data collection	From the students’ educational records, we collected their exam marks for each term for two consecutive academic years. The exam marks collected for 3 terms and for two academic years include Math, Science, and English. However, prior to admitting those students into the school, the corresponding records stored in the school that include entry exam marks, nationality, and schooling system they came from, were also collected. Halde [1] presented research on describing various ways in which machine learning is used in educational institutes and how they can get prediction of students’ performance. Halde [1] Addressed that student performance prediction can be made more precise by considering the learning style of students, their motivation and interest, concentration level, family background, personality type, information processing ability and the way they attempt exams, however the limitation is that the study has not considered students’ performance in exams in their previous and current academic stage.
Description of data collection	Following the Ethical approval from LJMU, and after receiving a letter of permission *(A “Letter of Permission” was sent to the school by the “Department of Education and Knowledge” in Abu Dhabi)* from the UAE Ministry of Education, the British and American school was contacted to arrange a meeting. The school principal granted access to the gatekeeper for data collection. The data from the British and American school was collected in excel format which was prepared by the gatekeeper. The dataset was updated periodically when needed.
Data source location	City/Town/Region: Mohammad Bin Zayed City, Abu Dhabi Country: United Arab Emirates City/Town/Region: Khalifa City A, Abu Dhabi Country: United Arab Emirates
Data accessibility	The dataset is published on Mendeley with DOI: 10.17632/3g8dtwbjjy.2 URL: http://dx.doi.org/10.17632/3g8dtwbjjy.2

## Value of the Data

• UAE is a multicultural country with many expats relocating from regions such as Asia, Europe and America. In order to meet expats needs, United Arab Emirates has established many international private schools. However, since every country has a different curriculum, many challenges were faced by schools and the Ministry of Education in allocating students to their correct year group and keeping track of their academic performance when relocating between schools and assigning student to their right level. Consequently, these data are important to show issues in student levelling faced by schools and MOE in different curriculums. Also, these data are useful for highlighting how students’ levels can vary when they transfer between curriculums.
• The dataset comprises novel aspects specifically, in terms of student grading in diverse educational cultures within the multiple countries – Researchers and other education sectors can use this data to see the impact of having varied curriculums in a country.
• The dataset can be used by the intelligent algorithms specifically machine learning and pattern analysis methods, to develop an intelligent framework applicable in multi-cultural educational systems to aid in a smooth transition “levelling”, hereafter of students who relocate from a particular education curriculum to another; and minimise the impact of switching on the students’ educational performance [2,3].

## 1 Data Description

**Fig. 1:**
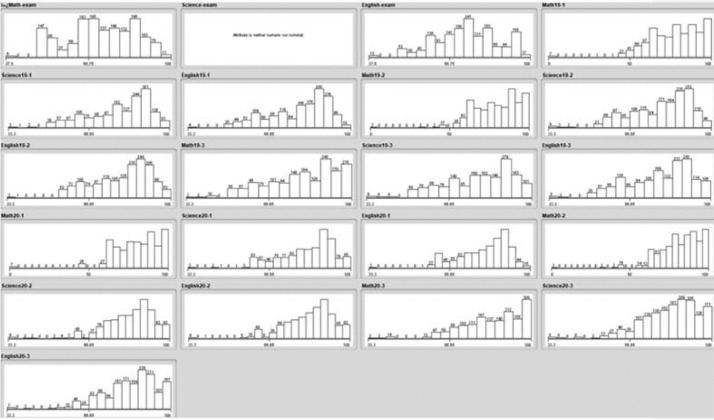
Data collection summary for exam marks.

**Fig. 2:**
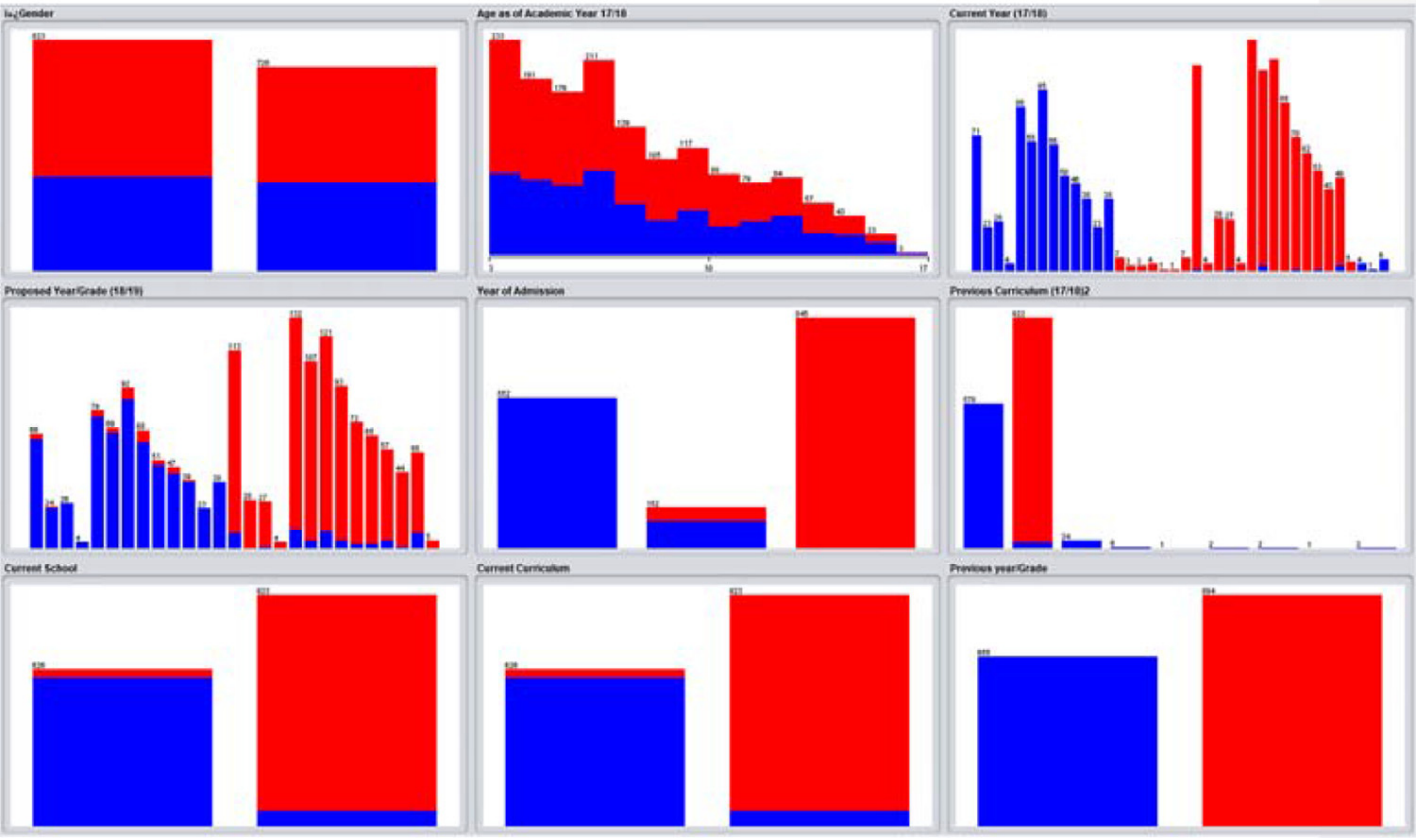
Data collection summary of general student information.

The dataset in Table 1 describes the attributes used to track student levelling in an international school based on three terms within three core subjects: Math, English, and Science, over two academic years. The collected dataset consists of 1550 records, which has columns (attributes) as illustrated in Table 1. A summary of the collected data with regards to student grades is shown in Fig. 1 while Fig. 2 shows a summary of general information of students.

**Table 1:** Collected data description.

Attribute Name	Value	Description
Gender	Male/Female	Gender of the student
Student Age (As of 2017/18)	6,7,8,9,…..etc.	Age of the student calculated from 2017/18 academic year
Proposed Year/Grade	Year 3, 4, 5 etc./Grade 3, 4, 5 etc.	This is the year or grade group assigned to the student by the school
Year of Admission	2017–18 /2018–19	The data collected is for two academic years *(an academic year is the period of the year during which students attend school),* 2017/18 academic year and 2018/19 academic year.
Previous Curriculum	British/American/MOE/Canadian/ Indian/Australian/CBSE/German	The curriculum the student transferred from.
Current Curriculum	British/American	The curriculum the student transferred to.
Previous Year/Grade	Year 3, 4, 5 etc./Grade 3, 4, 5 etc.	The year or grade the student was assigned to in his/her previous school
Math Entry Exam Mark	Mark out of 40	Exam marks for school entry exam in math
Science Entry Exam Mark	Mark out of 40	Exam marks for school entry exam science
English Entry Exam Mark	Mark out of 40	Exam marks for school entry exam English
Maths Marks 19–1 (2018/19, Term 1)	Percentage out of 100%	Term 1 student Maths Exam marks during academic year 2018/19
Science Marks 19–1 (2018/19, Term 1)	Percentage out of 100%	Term 1 student science Exam marks during academic year 2018/19
English Marks 19–1 (2018/19, Term 1)	Percentage out of 100%	Term 1 student English Exam marks during academic year 2018/19
Maths Marks 19–2 (2018/19, Term 2)	Percentage out of 100%	Term 2 student Maths Exam marks during academic year 2018/19
Science Marks 19–2 (2018/19, Term 2)	Percentage out of 100%	Term 2 student science Exam marks during academic year 2018/19
English Marks 19–2 (2018/19, Term 2)	Percentage out of 100%	Term 2 student English Exam marks during academic year 2018/19
Maths Marks 19–3 (2018/19, Term 3)	Percentage out of 100%	Term 3 student Maths Exam marks during academic year 2018/19
Science Marks 19–3 (2018/19, Term 3)	Percentage out of 100%	Term 3 student science Exam marks during academic year 2018/19
English Marks 19–3 (2018/19, Term 3)	Percentage out of 100%	Term 3 student English Exam marks during academic year 2018/19
Maths Marks 20–1 (2019/20, Term 1)	Percentage out of 100%	Term 1 student Maths Exam marks during academic year 2019/20
Science Marks 20–1 (2019/20, Term 1)	Percentage out of 100%	Term 1 student science Exam marks during academic year 2019/20
English Marks 20–1 (2019/20, Term 1)	Percentage out of 100%	Term 1 student English Exam marks during academic year 2019/20
Maths Marks 20–2 (2019/20, Term 2)	Percentage out of 100%	Term 2 student Maths Exam marks during academic year 2019/20
Science Marks 20–2 (2019/20, Term 2)	Percentage out of 100%	Term 2 student science Exam marks during academic year 2019/20
English Marks 20–2 (2019/20, Term 2)	Percentage out of 100%	Term 2 student English Exam marks during academic year 2019/20
Maths Marks 20–3 (2019/20, Term 3)	Percentage out of 100%	Term 3 student Maths Exam marks during academic year 2019/20
Science Marks 20–3 (2019/20, Term 3)	Percentage out of 100%	Term 3 student science Exam marks during academic year 2019/20
English Marks 20–3 (2019/20, Term 3)	Percentage out of 100%	Term 3 student English Exam marks during academic year 2019/20

The school name, student name and ID are eliminated as per ethical approval.

## 2 Experimental Design, Materials and Methods

**Fig. 3:**
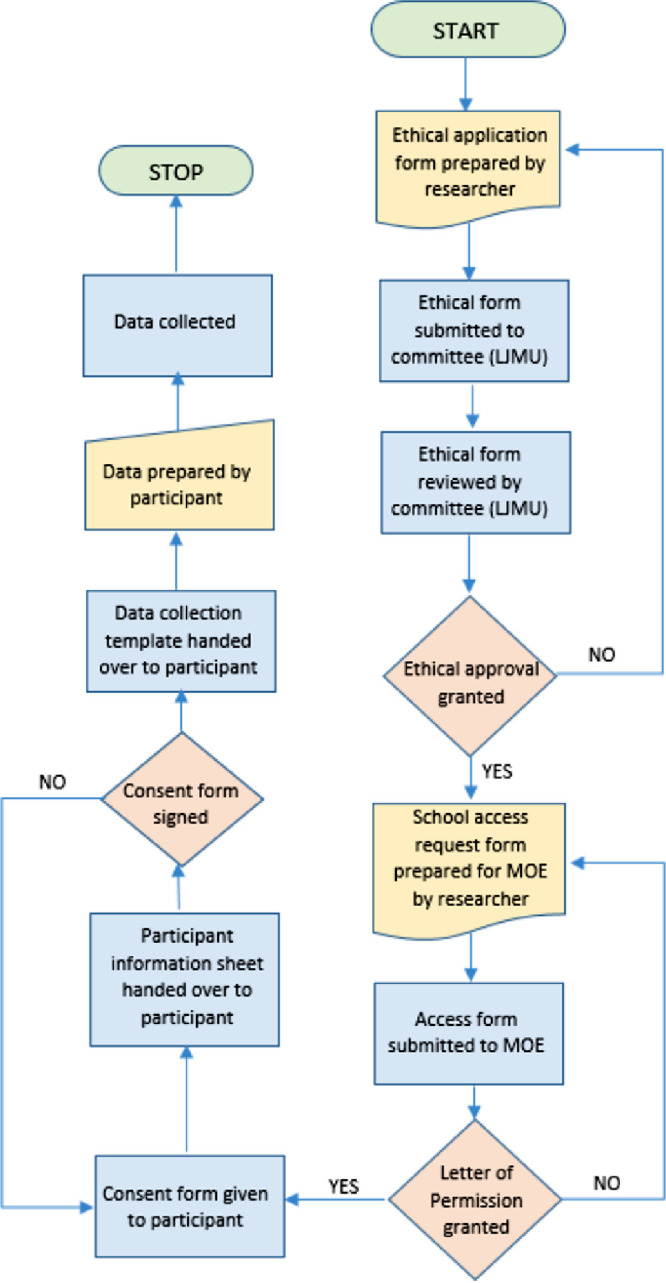
Data collection process.

Fig. 3 demonstrates the data collection process used in this study. The collected data was in the form of excel sheet which the gatekeeper was recruited to provided actual student data. A “Letter of Permission” (as per the procedures provided by the Department of Education and Knowledge in Abu Dhabi) from the Department of Education and Knowledge (that is addressed to the school principal) was sent requesting for permission to collect data within the school. Written consent was required by the participants to allow the researcher to conduct the research which was given directly to the participants alongside the participant information sheet which had all contact information about the researcher. The written consent forms are taken directly from the participants. As the age of the students are young, the data requested from the school does not have any personal information about the students, and in order to ensure there's no personal data released, the gatekeeper shared data as per the written consent form.

Technical validation of data is an essential part when handling data which helps to check for correctness, meaningfulness, and the security of data to be used [4]. Therefore the data was prepared by unifying the outputs, filling missing data, eliminating non-related features, and preparing headers. Confidential data such as student name and their identification number was removed in order to remain compliance with our ethical approval.

## Ethics Statement

The dataset is collected through informed consent from all participants and gatekeeper. Permission from Ministry of Education and Knowledge is obtained. We obtained the ethical approval for this study from LJMU (19/CMS/001).

## Declaration of Competing Interest

None.
